# Comparison of Body Mass Index and fat percentage criteria classification of 7–13 year-old rural boys in South Africa

**DOI:** 10.1186/s12887-020-02419-9

**Published:** 2020-11-17

**Authors:** Maya van Gent, Anita Pienaar, Habib Noorbhai

**Affiliations:** 1 Department of Human Movement Sciences, Faculty of Health Sciences, University of Fort Hare, Alice, South Africa; 2 School for Human Movement Sciences, Faculty of Health Sciences, North West University, Potchefstroom, South Africa; 3 Department of Sport and Movement Studies, Faculty of Health Sciences, University of Johannesburg, Johannesburg, South Africa

**Keywords:** Anthropometry, Children, Body Composition, South Africa

## Abstract

**Background:**

The aim of this paper was to investigate whether BMI and fat percentage classification criteria, would classify a sample of 7–13 year old boys from a rural background in similar nutritional categories.

**Methods:**

A cross-sectional study with a stratified random sampling included 601 rural boys (7–13 years old). Fat percentage criteria classification and BMI were calculated and compared. Maturity status, and age at peak height velocity (PHV) were indirectly determined. Statistical techniques included descriptive statistics, Pearson product correlation coefficients, the Kappa agreement test and the McNemar’s test. The level of statistical significance was set at *p* ≤ 0.05.

**Results:**

All age groups presented with statistically significant high correlations between BMI and fat percentage, and low to medium correlations between fat percentage and maturity age (MA). Measurement of agreement between BMI and fat percentage classifications showed poor to fair agreements for all age groups, with the exception of the eight-year old group which presented a moderate agreement.

**Conclusions:**

Classifications based on BMI and fat percentage, results in different classifications for the same population. Until further research has been done to determine the best classification for nutritional status, it is recommended that both classification methods be used for more accurate classification of nutritional status.

## Background

Appropriate and valid nutritional classification using body composition assessments, are important for the early identification and treatment of malnutrition in children. Developing countries, such as South Africa, face a double burden of malnutrition, (where underweight and overweight can be found in one household) [1, 2]. Under nutrition (stunting and wasting) is linked more to lower socio-economic status and found mostly in rural areas [3]. Monyeki et al. [4] indicated that researchers reported high incidences of underweight, stunting and wasting among South African children, even more among boys from rural areas. Studies conducted among rural boys in South Africa have also reported increases in the prevalence of overweight and obesity [5–7]. Malnutrition was assessed in these studies by making use of the Body Mass Index (BMI). This method, and the interpretation of results related to BMI in children, has, however, been questioned by some researchers [8, 9].

The biggest criticism against using BMI to assess overweight and obesity is that it also reflects fat-free mass [10] and, therefore, cannot be considered an accurate indicator of body fat mass [11] and subsequently can contribute to misdiagnosis of malnutrition among children [12–14]. The World Health Organisation (https://www.who.int/growthref/who2007_bmi_for_age/en/) and the Centre for Disease Control and Prevention (https://www.cdc.gov/nccdphp/dnpao/growthcharts/training/bmiage/page4.html) have specific cut-off values to define overweight and obesity using BMI as reference, however Roubenoff et al. [9] suggests that BMI inadequately predicts the percentage of body fat, while Craig et al. [8] suggests that BMI based assessments of body fatness tend to be conservative compared to other body composition methods. More recent studies suggest that to combat overweight and obesity in children and adolescents, emphasis should rather be placed on assessing fat mass, and that the use of BMI as an indicator of the nutritional status should be used with caution [10, 13, 15].

Biological maturation of different bodily systems tends to proceed independently of chronological (calendar) age, and therefore, chronological age is not an adequate indicator of biological maturity [16, 17]. Early biological maturation (a process of predetermined genetic changes in form and complexity of bodily organs which take place as a result of human growth and which results in higher functioning of the neurological and physical systems) seems to influence the onset of weight in young children [18]. Early changes in biological maturation are associated with a greater prevalence of overweight and obesity when using BMI as the method of assessment [16, 17]. Ribeiro et al. [16] also found early maturation to be a risk factor associated with obesity among males, while Benedet et al.[17] reported a lower prevalence of obesity among late maturing males. As such, it could be suggested that the onset of the growth spurt (a parameter of the growth curve which signifies the reaching of puberty and which is considered to be a general indicator of maturity), and the wide variance among different ethical groups (particularly among the African cohort), might influence nutritional classification when using BMI [18, 19]. Sampei et al. [20] furthermore states that differences in body composition can exist between different ethnic groups within the same population and that this could be due to maturation status but also the wide variability in the onset of the growth spurt.

Sampei et al. [20] found age related correlations between BMI and other classification methods, where in 10–11 year old Japanese and Caucasian girls, the BMI correlated well with other classification methods of obesity. However, it was also reported in the same study that among 16–17 year old girls, the BMI presented low to no agreements with any other methods of determining obesity [20]. This suggests that timing of maturity might be a factor that influences the method of classification used when classifying obesity, at least in girls. The question, however, is whether this would be the case for boys living in rural areas of South Africa? In South Africa, it has been reported that rural boys enter puberty later [21], and as international studies seem to suggest, those who mature at a later stage, tend to show a lower rate of obesity [16, 17], which is in contradiction with some of the nutritional studies published in South Africa [5–7]. Therefore, the aim of the study was to compare BMI and fat percentage criteria classifications of underweight, normal weight, overweight and obesity among rural boys, aged 7–13 years, residing in the Eastern Cape, South Africa.

## Methods

### Study design

This was a cross-sectional study and formed part of a bigger study titled “Evaluation of the national school nutrition programme and the Tiger Brands Foundation in-school breakfast programme in the Lady Frere and Qumbu district of the Eastern Cape, South Africa” [22]. This study was a joined project between Tiger Brands Foundation, the Centre for Social Development in Africa at the University of Johannesburg and the University of Fort Hare, South Africa.

### Sample

A sample of 358 boys (aged 7–13 years) from secondary, combined and primary schools in the Lady Frere district and a further 243 in the Qumbu district of the Eastern Cape were included in the study. Written consent was obtained from all participants and their parents. Lists of all the schools per district (Lady Frere and Qumbu) were obtained from the Eastern Cape Department of Basic Education, South Africa. Stratified random sampling was used to select the participating schools per district. A criterion for the inclusion of the schools was that they had to fall within Quantile 1–3 of The Amended National Norms and Standards for School Funding (ANNSSF) used by the National Department of Basic Education in South Africa. Quantile 1–3 schools are schools that represent very poor to poor economic sectors from the same geographical environment. Stratified random sampling was used for the study to ensure that there was an adequate representation of schools at the 95% confidence level with a 5% margin for error. A total of 41 schools were identified. Within each school, stratified sampling by grade and gender was used to determine which students would participate in the study. Once the data set was cleaned, the final sample included 602 boys (aged 7–13 years). This provided a 2.57% margin for error at the 95% confidence level.

### Instruments and measurements

All anthropometric measurements were taken according to the ISAK protocol (International Society for the Advancement of Kinanthropometry) [23] accredited Level 1 ISAK anthropometrists and in accordance with assessments of anthropometric measurements [24]. Participants were measured with minimal clothing and no shoes. Body mass was measured to the 0.1 kg by making use of a SECA electronic scale. Body stature was measured as the perpendicular distance between the transverse planes of the *Vertex* and the inferior aspect of the feet, to 0.1 cm. The BMI was calculated by dividing body mass with stature (kg/m²). BMI values were classified according to International cut off points for thinness (grade1, 2, 3), overweight and obesity according to gender and age (2–18 years) [25, 26]. BMI values were classified according to International cut off points for thinness (grade1, 2, 3), overweight and obesity according to gender and age (2–18 years) [8, 25, 26]. The BMI classification for thinness, overweight and obesity for seven-year old boys (≤ 14.04; 17.92; 20.63), eight-year old boys (≤ 14.15; 18.44; 21.60), nine-year old boys (≤ 14.35; 19.10; 22.77), 10-year old boys (≤ 14.64; 19.84; 24.00), 11-year old boys (≤ 14.97; 20.55; 25.10), 12-year old boys (≤ 15.35; 21.22; 26.02) and 13-year old boys (≤ 15.84; 21.91; 26.84) were used. All other BMI values obtained not in the cut off values mentioned were classified as normal.

Sitting height was measured as the perpendicular distance between the transverse planes of the *Vertex* and the inferior aspects of the buttocks when seated, to 0.1 cm. Arm span was measured as the perpendicular distance between the *dactylia* on the left and right arms with the arms outstretched horizontally, to 0.1 cm.

The triceps and calf skinfolds measures were taken using an Innovare skinfold calliper and were recorded to the nearest 0.5 mm. The triceps skinfold was taken parallel to the long axis of the arm at the triceps skinfold site (posterior surface of the arm, in the mid-line, at the level of the *Mid-acromiale-radiale* landmark), while the medial calf skinfold measurement was taken vertically at the medial calf skinfold site (the point on the most medial aspect of the calf at the level of the maximum girth). All measurements were taken twice, and in the event that the first two measurements differed with more than 0.5 cm/kg for the body mass and stature, and 0.5 mm for skinfolds, a third measurement was taken. The first two skinfold measurements were used to calculate the average skinfold thickness, which was then used for data analysis. The sum of skinfolds consisted of triceps and calf skinfolds. These skinfold measurements were also used to calculate fat percentage, by making use of Slaughter et al. [27] fat percentage charts for boys. Fat percentage criteria classification was done for low adiposity (≤ 10 mm), normal adiposity (11–25 mm) and high adiposity (≥ 26 mm) [27].

Chronological age of the participant was calculated by subtracting the date of birth from the date of measurement, and then classified in one-year intervals, for example, seven-year-old boys would be classified from 6.5 to 7.49 years. Table 1 indicates the total number of participants per age group as well as the ranges for each age group. Estimated timing of maturity was defined by the timing of the adolescent growth spurt (a period in time in which a child experiences their fastest upward growth in stature during adolescence, also known as peak height velocity). The onset of growth spurt coincides also with the onset of maturity [18].

**Table 1:** The Anthropometrical measurements of 7–13 year old rural boys (mean ± SD, range)

Anthropometrical measures	7 year old *n* = 71	8 year old *n* = 82	9 year old *n* = 93	10 year old *n* = 100	11 year old *n* = 101	12 year old *n* = 82	13 year old *n* = 73
**Chronological age (years)**	7.04 ± 0.29 6.51–7.49	8.06 ± 0.26 7.54–8.49	9.02 ± 0.30 8.52–9.47	9.97 ± 0.28 9.50-10.49	11.08 ± 0.27 10.53–11.49	11.92 ± 0.30 11.50-12.49	13.00 ± 0.29 12.51–13.49
**Body mass** **(kg)**	22.89 ± 2.52 22.29–23.48	24.71 ± 2.68 24.13– 25.30	27.02 ± 4.79 26.04–28.01	29.43 ± 5.20 28.32–30.46	32.88 ± 4.92 31.90-33.85	35.28 ± 6.41 33.87–36.69	39.70 ± 6.95 38.08–41.33
**Body stature (cm)**	119.32 ± 4.30 118.30–120.33	124.29 ± 4.69 123.26-125.32	128.97 ± 5.99 127.73–130.20	132.91 ± 5.74 131.78-134.05	138.31 ± 5.81 137.16-139.46	141.99 ± 7.22 140.40-143.58	149.29 ± 7.67 147.50-151.08
**BMI** **(kg/m²)**	16.05 ± 1.28 15.75–16.35	15.98 ± 1.30 15.69–16.26	16.19 ± 2.10 15.76–16.62	16.58 ± 2.01 16.18–16.98	17.13 ± 1.84 16.76–17.50	17.39 ± 2.19 16.91–17.87	17.73 ± 2.03 17.20-18.27
**Triceps skinfold (mm)**	6.79 ± 2.28 6.24–7. 32	6.82 ± 1.72 6.44–7.20	6.62 ± 3.02 6.00-7.25	7.24 ± 2.83 6.67–7.79	7.91 ± 3.56 7.21–8.62	8.35 ± 4.00 7.47–9.23	8.18 ± 4.07 7.24–9.13
**Calf skinfold (mm)**	6.90 ± 2.48 6.31–7.48	7.30 ± 2.24 6.82–7.80	7.21 ± 4.11 6.37–8.06	8.11 ± 3.98 7.32–8.90	8.99 ± 4.62 8.07–9.90	9.84 ± 5.45 8.64–11.04	9.58 ± 4.63 8.50-10.66
**Sum of skinfolds (mm)**	13.68 ± 4.29 12.76–14.70	14.12 ± 3.47 13.36–14.89	13.84 ± 6.84 12.43–15.25	15.35 ± 6.35 14.08–16.61	16.90 ± 7.69 15.38–18.42	18.20 ± 8.80 16.26–20.13	17.76 ± 8.13 15.87–19.66
**Fat (%)**	9.34 ± 2.62 8.72–9.97	9.61 ± 2.19 9.15–10.08	9.44 ± 4.17 8.58–10.30	10.36 ± 3.88 9.59–11.13	11.31 ± 4.70 10.38–12.24	12.10 ± 5.37 10.92–13.23	11.84 ± 4.96 10.68-13.00
**Predicted age at PHV (years)**	12.40 ± 0.25 12.33–12.45	12.91 ± 0.22 12.87–12.96	13.35 ± 0.32 13.28–13.42	13.78 ± 0.33 13.71–13.84	14.25 ± 0.34 14.19–14.32	14.62 ± 0.42 14.53–14.71	14.92 ± 0.47 14.81–15.03
**Estimated maturity timing (years)**	5.35 ± 0.23 5.29–5.40	4.85 ± 0.25 4.80–4.91	4.32 ± 0.30 4.27–4.39	3.80 ± 0.36 3.72–3.87	3.16 ± 0.377 3.09–3.24	2.70 ± 0.44 2.60–2.79	1.92 ± 0.511 1.80–2.04

Estimated maturity timing was determined by means of Maturity offset (the age at which a child is expected to achieve PHV) for each participant. This estimation was done by making use of the equation of Mirwald et al. [24] as follows: Maturity Offset = -9.236 + 0.0002708·Leg Length and Sitting Height interaction − 0.001663·Age and Leg Length interaction + 0.007216·Age and Sitting Height interaction + 0.02292·Weight by Height ratio, where *R* = 0.94, *R*²= 0.891, and SEE = 0.592. Those participants who matured early were classified with a maturity offset less than − 0.50 (before maturity offset ) while children who fell between − 0.49 and 0.48 were classified as those who matured averagely (during maturity offset ), while boys with a maturity age of more than 0.49 were classified as those who matured late (after maturity offset) [28].

### Data analyses and statistical methods

Descriptive statistics were calculated by using SPSS 23 for all variables and the mean, standard deviation and the range (-95 and 95% Confidence levels) are reported in Table 1. Before subsequent tests were performed, normality of the data was verified using the Shapiro-Wilk test. Frequency statistics were used to establish the percentage classification in the BMI and fat percentage group criteria’s.

The Pearson product correlation matrix was used to establish relationships between BMI, Fat percentage and Maturity age. Correlation values were classified as low (*r* = 0.1), medium (*r* = 0.3) and high (*r* = 0.5) [29]. Lastly, the agreement of methods between the BMI and Fat percentage classification was statistically evaluated by making use of the Kappa agreement test [30]. The McNemar’s test [31] was used to assess the significance of the differences between the two correlated proportions (according to the BMI and fat percentage classifications). The level of statistical significance was set at *p* ≤ 0.05. Kappa Strength of agreement was classified as poor = < 0.00; slight = 0.00-0.20; fair = 0.21–0.40; moderate = 0.41–0.60; substantial = 0.61–0.80 and almost perfect = 0.81-1.00.

## Results

Table 1 represents the descriptive statistics for 7–13 year old boys.

The relative technical error of measurements (TEM) for all anthropometrical measurements were calculated according to Intra-evaluator TEM calculations [32]. The TEM ranges from 1.2% (body mass) to 6.7%, (Triceps skinfold), all of which are still acceptable.

As expected, stature and body mass increased with increasing age. The lowest BMI value was found for the eight-year old boys (15.98 ± 1.30) and this group only presented the third lowest fat percentage (9.61 ± 2.19) while the seven-year olds (9.34 ± 2.62) and nine-year olds (9.44 ± 4.17) had lower fat percentages. The 13-year old boys presented with the highest BMI values (17.73 ± 2.03), the second highest fat percentage (11.84 ± 4.96) and the lowest maturity age (1.92 ± 0.51), which indicates that they were the closest to reaching their PHV (an estimated indicator of maturation status). Another expected result was the maturity age which showed to be the highest for the seven-year olds (5.35 ± 0.23) as they were the furthest away from PHV. It is clear from Table 1 that all boys in this study were late developers in terms of reaching maturation.

Table 2 presents the percentage occurrence of participants in each criterion for BMI and fat percentage classifications. The highest percentage of the group, when using BMI as the classification criteria, was present in the normal weight category among all the ages. However, when using fat percentage as the classification criteria, the highest occurrence of participants were found in the low adiposity groups for seven to 10-year olds, and in the normal adiposity category for 11–13 year olds. It was also noted that no occurrence was found in the high adiposity category for 7–8 year old boys, while 5.6-4.9% was reported in the overweight/obesity category based on BMI for the same age group. The occurrence of high adiposity classification and overweight/obesity classification are however, almost similar for 9 to 13 year of boys. The percentage occurrence, in the low adiposity category, ranged from 52–59.2% for 7–13 year old boys, while the percentage occurrence in the BMI thinness category is much lower, ranging from 3–12.3%. The most noticeable difference between these two criteria for classification seems to apply to the normal weight and thinness categories (in BMI) and the low-and normal adiposity (fat percentage) categories.

**Table 2:** Percentage occurrence of BMI and Fat percentage classification criteria of 7–13 year old rural boys

Age groups	Body mass index (BMI)			Fat percentage		
	Thi %	NW %	OW/OB %	LA %	NA %	HA %
**7 year old**	5.6%	88.7%	5.6%	59.2%%	40.8%	0%
**8 year old**	6.1%	89.0%	4.9%	58.5%	41.5%	0%
**9 year old**	9.7%	87.1%	3.2%	65.6%	31.2%	3.2%
**10 year old**	6.0%	91.0%	3.0%	52%	45.0%	3.0%
**11 year old**	3.0%	94.0%	3.0%	43.6%	50.5%	5.9%
**12 year old**	9.8%	81.7%	8.5%	42.7%	47.6%	9.8%
**13 year old**	12.3%	83.6%	4.1%	42.5%	50.7%	6.8%

*Thi* Thinness, *NW* normal weight, *OW/OB* overweight/ obese, *LA* Low adiposity, *NA* normal adiposity, *HA* high adiposity

Table 3 represents the relationship between BMI, fat percentages and maturity age. Correlating BMI and fat percentage, the researchers controlled for maturity age (MA) as to exclude the variable’s influence from the correlation. Most of the age groups, with exception of the eight-year olds, presented high positive and significant correlations (*r* ≥ 0.50) (*p* ≤ 0.05) between BMI and fat percentage. The correlation between BMI and MA presented significant low (*r*=-0.23) to medium (*r*=-0.46) negative correlations for the 7–9 and 13-year old boys, while high significant negative correlations were found for 10-12-year-old boys (*r*=-0.51; *r*=-0.52; *r*=-0.64). The correlation between fat percentage and MA presented similar tendencies that were observed between BMI and MA. However, the only age group that presented high significant negative correlations were the nine-year old (*r*=-0.48) and 12-year old (*r*=-0.49) boys. This seems to suggest that there is less pronounced relationship between MA and fat percentages, compared with MA and BMI.

**Table 3:** Correlation between Body Mass Index (BMI), Fat percentage (Fat %) and Maturity Age (MA) for 7–13 year old boys (controlled for Maturity Age (MA))

Age groups	BMI vs. Fat % *(Controlling for MA)*		BMI vs. MA		Fat % vs. MA	
	***r-value***	***p-value***	***r-value***	***p-value***	***r-value***	***p-value***
**7 year old**	0.69†	0.00*	-0.23	0.00*	-0.20	0.10
**8 year old**	0.45†	0.00*	-0.36	0.00*	-0.32	0.04*
**9 year old**	0.75†	0.00*	-0.44	0.00*	-0.48†	0.00*
**10 year old**	0.77†	0.00*	-0.51†	0.00*	-0.39	0.00*
**11 year old**	0.70†	0.00*	-0.52†	0.00*	-0.33	0.01*
**12 year old**	0.71†	0.00*	-0.64†	0.00*	-0.49†	0.00*
**13 year old**	0.54†	0.00*	-0.46†	0.00*	-0.25	0.03*

Low correlation *r* = 0.1; medium *r* = 0.3; high correlation *r* = 0.5†; statistical significance *p ≤ 0.05

The Kappa [30] and McNemar [31] analysis was done to determine the agreement between the classification criteria for BMI and fat percentage and is presented in Table 4. The seven-year old boys presented with a poor measure of agreement (κ =-0.26), while the nine to 11-year old boys presented with a slight measure of agreements (κ = 0.11 to κ = 0.18). The 12 and 13-year old boys presented with fair measures of agreements (κ = 0.22 to κ = 0.30). The highest measure of agreements was found for the eight-year old boys (κ = 0.57), which was however still classified as moderate measure of agreement. The McNemar test [31] that was used to determine the differences between the paired proportion (in other words the proportions of how the children were classified according to BMI and fat percentage categories) indicated significant differences between these two classification methods.

**Table 4:** Kappa and McNemar measure of agreement between BMI and Fat percentage categorise for 7–13 year old rural boys

Age groups	Measure of agreement Kappa		McNemar test for significance
	**Kappa (κ)**	**SoA**	
**7 year old**	-0.26	Poor	0.00*
**8 year old**	0.57	Moderate	0.00*
**9 year old**	0.14	Slight	0.00*
**10 year old**	0.18	Slight	0.00*
**11 year old**	0.11	Slight	0.00*
**12 year old**	0.22	Fair	0.00*
**13 year old**	0.30	Fair	0.00*

*SoA* Strength of agreement, Kappa Strength of agreement: Poor = < 0.00; Slight = 0.00-0.20; Fair = 0.21–0.40; Moderate = 0.41–0.60; Substantial = 0.61–0.80; Almost perfect = 0.81-1.00

Significance = *p ≤ 0.05

## Discussion

The main aim of this study was to compare the level of agreement between BMI and fat percentage criteria for classification of underweight, normal weight, overweight and obesity among of 7-13-year old boys that grow up in rural area in the Eastern Cape Province, South Africa. The results indicated significant correlations between BMI and fat percentage, while both measures also correlated moderately and higher with MA, but the level of agreement between these classification methods were found to be questionable. However, the significant high correlation between BMI and fat percentage for all age groups that were found is aligned with previous studies [10, 12, 13]. It was however noted that higher correlations were found between BMI and MA in more age groups, compared to correlations between fat percentage and MA. This might imply that MA has a stronger relationship to BMI compared to fat percentage, which could possibly influence the classification criteria. Some studies have also found associations between obesity and maturation, although not using the indirect method, when BMI was used as a classification criterion [16, 17]. Not many studies could be found that used this indirect method of determining maturity and comparing it to body composition. One such study of Juliano-Burns et al. [33] conducted among adolescents, found that late maturing boys had more bone mineral density, more lean mass and were taller at the age of PHV compared to those who matured early. Seeing that all the boys in this study were maturing late, it might explain the high negative correlations between BMI and maturity age. The high lean mass could possibly influence the criteria classification according to BMI, as previous studies have highlighted this as a possible limitation of BMI [10, 13, 14]. Unfortunately, no studies could be found that reported any association between maturation and fat percentage (using the skinfold method).

The findings should be considered against the background of the following limitations of the study. The first shortcoming that should be highlighted is that maturity status was calculated indirectly and, therefore, limited studies are available that used this method to compare our findings. The method used to estimate PHV might not be applicable to this sample for two main reasons; firstly, this sample consisted only of 7-13-year old boys and hence the occurrence of the onset of growth spurt could have happened later, which would not be indicated when using this method. Secondly, the original paper of Mirwald et al. [24] cautioned that when the equation is applied to other samples, there may be a loss of accuracy in predicting maturity offset. The accuracy of the equation is also reported to be the highest between 12 and 16 years in males [24] and this seems to be the case for this study. Another possible limitation with this equation is that equations in the study have major limitations with early and late maturing boys and girls [34]. This should also be considered for the current study.

Nonetheless, our findings of the 7-13-year old boys maturing late is still in line with findings by Cole et al. [26] who also found that rural African boys tend to enter puberty later compared to other ethnic groups. A possible explanation for this could be that these boys come from very poor surroundings, which would result in some nutritional deficiencies. It is well documented that nutritional status is also known to affect the timing of puberty (rough index of maturation) [16, 35] and that this could explain why all the boys were classified as reaching puberty late.

The second shortcoming is, that no “gold standard” method for assessing body composition was used, and it therefore cannot be concluded which of the methods used is more appropriate for this specific population. Nevertheless, the results present some correlations between BMI, fat percentage and MA as well as comparing the BMI and fat percentage classification criteria’s.

The overweight and obesity occurrence among the 7-13-year old boys (as derived from Table 2) were much in accordance with other studies done in South Africa It was reported that among 6–13 year old boys, 14.0% were overweight and 3.2% obese [7]. However, this study included all race groups from various socio-economical environments. It was also reported in another study that 6.4% were overweight and 3.3% had obesity among 6-7-year old children from Quantile 1–3 schools in the North-West province [36]. Although one study [17] reported a lower prevalence of overweight and obesity among late maturing males, it seems in this case that boys who mature earlier are still classified as having high occurrences of overweight/ obesity. None of the boys in this present study presented with severe thinness, however, small percentages of thinness were still observed in grade 1–3, as determined by Cole et al. [26].

The strong point of this study is, however, that it provides more understanding of different assessment methods of body composition characteristics and how estimated timing of maturity in boys growing up in rural areas in South Africa will influence body composition as very few studies have been conducted in this age group on children in South Africa [37]. The development of indirect, cost effective and non-intrusive methods to determine maturity, could provide valuable information with regards to nutritional status for various populations, even more so for developing countries. Future studies should also include longitudinal research that might look at the impact of other factors such as socio- economic status and nutritional status of the population. More research is also encouraged on same aged children from urban areas in South Africa.

## Conclusions

Differences in the classification criteria of BMI and fat percentage for the 7-13-year old boys were evident from this study. The strength of agreement between BMI and fat percentage criteria classification was found to be poor to moderate, indicating that these two methods of criteria classification do not classify all the same boys between 7 and 13 years that grow up in rural areas in similar nutritional classifications. Although it cannot be said which of these classification criteria’s are more appropriate for this population, it does warrant further investigation and some suggestions should be considered. Firstly, even though the use of BMI is acknowledged as cost effective and requiring low skill levels to administer, the limitation is that a measure of heaviness rather than fatness cannot be ignored.

Alternative methods should, however, also be investigated or developed that focus more on adiposity while considering the influence of maturity. Some studies have investigated the height-to-waist ratio in this regard as a possible nutritional classification index [38–40] but again only a few of these studies included rural populations in their analysis. Secondly, future research should investigate the longitudinal influence of maturity (directly measured) on various body composition components among rural populations. Until such research has been conducted, it is recommended that both methods (fat percentage and BMI) be used for a more accurate classification of nutritional status. Boys at risk of being overweight or obese, could be more closely monitored by evaluating other factors such as direct measurement of maturity, nutritional intake, socio-economic status and physical activity levels.

## Data Availability

All data generated or analysed during this study available upon request.

## Abbreviations

BMI: Body Mass Index
PHV: Peak Height Velocity
MA: Maturity Age
ISAK: International Society for the Advancement of Kinanthropometry
